# Chronic Supplementation of Paeonol Combined with Danshensu for the Improvement of Vascular Reactivity in the Cerebral Basilar Artery of Diabetic Rats

**DOI:** 10.3390/ijms131114565

**Published:** 2012-11-08

**Authors:** Jing Hu, Ya-Ling Li, Zi-Lin Li, Hua Li, Xuan-Xuan Zhou, Peng-Cheng Qiu, Qian Yang, Si-Wang Wang

**Affiliations:** 1 Institute of Materia Medica, School of Pharmacy, Fourth Military Medical University, Xi’an 710032, China; E-Mails: hujinglzbx@163.com (J.H.); lihua199@yeah.net (H.L.); XXZ19977@163.com (X.-X.Z.); qpc022@126.com (P.-C.Q.); yanqian279@163.com (Q.Y.); 2 General Hospital of Lanzhou Command, PLA, Lanzhou 730050, China; E-Mail: lizilin98@chinaren.com; 3 Department of Special Diagnosis, The Second Authority Clinic of Lanzhou Command, PLA, Lanzhou 730000, China; E-Mail: liyaling65@163.com

**Keywords:** paeonol, danshensu, vasodilation, endothelium dysfunction, vascular smooth muscle cell, oxidative stress, Ca^2+^ channel

## Abstract

One of the leading causes of death in the world is cerebrovascular disease. Numerous Chinese traditional medicines, such as Cortex Moutan (root bark of *Paeonia suffruticosa* Andrew) and *Radix Salviae miltiorrhizae* (root and rhizome of *Salvia miltiorrhiza* Bunge), protect against cerebrovascular diseases and exhibit anti-atherosclerotic effects. Traditional medicines have been routinely used for a long time in China. In addition, these two herbs are prescribed together in clinical practice. Therefore, the pharmacodynamic interactions between the active constituents of these two herbs, which are paeonol (Pae) and danshensu (DSS), should be particularly studied. The study of Pae and DSS can provide substantial foundations in understanding their mechanisms and empirical evidence to support clinical practice. This study investigated the effects and possible mechanisms of the pharmacodynamic interaction between Pae and DSS on cerebrovascular malfunctioning in diabetes. Experimental diabetes was induced in rats, which was then treated with Pae, DSS, and Pae + DSS for eight weeks. Afterward, cerebral arteries from all groups were isolated and equilibrated in an organ bath with Krebs buffer and ring tension. Effects of Pae, DSS, and Pae + DSS were observed on vessel relaxation with or without endothelium as well as on the basal tonus of vessels from normal and diabetic rats. Indexes about oxidative stress were also determined. We report that the cerebral arteries from diabetic rats show decreased vascular reactivity to acetylcholine (ACh) which was corrected in Pae, DSS, and Pae + DSS treated groups. Furthermore, phenylephrine (PE)-induced contraction response decreased in the treated groups. Phenylephrine and CaCl_2_-induced vasoconstrictions are partially inhibited in the three treated groups under Ca^2+^-free medium. Pre-incubated with tetraethylammonium, a non-selective K^+^ channel blocker, the antagonized relaxation responses increased in DSS and Pae + DSS treated diabetic groups compared with those in diabetic and Pae-treated diabetic groups. In addition, superoxide dismutase activity and thiobarbituric acid reactive substances content significantly changed in the presence of Pae + DSS. We therefore conclude that both Pae and DSS treatments prevent diabetes-induced vascular damage. Furthermore, Pae + DSS prove to be the most efficient treatment regimen. The combination of Pae and DSS produce significant protective effects through the reduction of oxidative stress and through intracellular Ca^2+^ regulatory mechanisms.

## 1. Introduction

Shuang-Dan prescription combines the use of Cortex Moutan (root bark of *Paeonia suffruticosa* Andrew) and *Radix Salviae miltiorrhizae* (root and rhizome of *Salvia miltiorrhiza* Bunge), which are famous herbs widely used in traditional Chinese medicine. In clinical practice, the Shuang-Dan prescription is often used for treating cerebrovascular and cardiovascular diseases. However, the prescription contains a complex mixture of compounds. In addition, some of the compounds in the whole prescription have redundant pharmacological effects. Therefore, the prescription is still not extensively accepted in the western world. Simplifying the constitution and elucidating the prescription’s mechanisms should be the primary concern.

**Figure 1:**
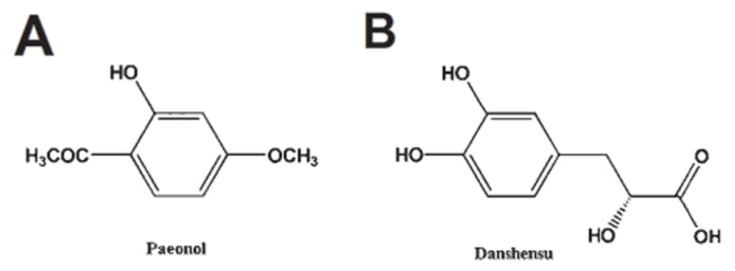
Chemical structures of Pae (**A**) ([2R]-3-[3,4-di-hydroxyphenyl]-2- hydroxypropanoic acid) and DSS (**B**) (4-methoxy-2-hydroxyacetophenone).

Paeonol (Pae, Figure 1, 20-hydroxy-40-methoxyacetophenone) is a major phenolic component in Cortex Moutan [1–4], whereas danshensu (DSS, Figure 1, 3-(3,4-dihydroxyphenyl) lactic acid) is a water-soluble active component isolated from *Radix Salviae miltiorrhizae*[5–7]. Similar to other natural compounds [8], several studies showed that Pae and DSS elicit an endothelium-independent relaxation of isolated rat aorta [9,10] and protection of endothelial cells [11,12], respectively. We previously reported that the combined use of Pae and DSS has synergistic protective effects on focal cerebral ischemia-reperfusion injury in rats [13]. Moreover, Pae combined with other hydrophilic phenolics of *Radix Salviae miltiorrhizae* could attenuate oxidative stress, protect vascular functions [14], and synergistically protect against myocardial ischemia in rabbits [14]. Recently, we found that the co-administration of DSS increases the concentration of Pae in heart and brain tissues [15] and increases pharmacological activity in treating cerebrovascular and cardiovascular diseases [16]. However, the mechanism of the interactions of representative active components in the protection of vascular function is not well understood.

Diabetes mellitus (DM) causes multiple dysfunctions such as vascular dysfunction, which increases the risk of stroke. Vascular dysfunctions are one of the major causes of morbidity and mortality in patients with DM. Previous studies reported that forearm blood flow responsive to acetylcholineis reduced in type 2 diabetes, suggesting endothelial dysfunction [17,18]. Moreover, vascular smooth muscle (VSMC) exhibits hyper-reactivity, hypertrophy and apoptosis in diabetes [19–23]. One of the pathogenesis of diabetic vascular dysfunction is oxygen derived free radicals, which are significantly elevated under DM [24–26]. Diabetic vascular dysfunction is also related to increased Ca^2+^ influx [27] and inhibited vascular K^+^ channels [28]. Previous studies showed that the inhibition of vascular K^+^ channels increases Ca^2+^ influx, which leads to depolarization and vasoconstriction [28].

Therefore, the aim of this study is to investigate the effects of Pae + DSS on diabetes-induced dysfunction of cerebral arteries compared with the individual effects of Pae or DSS.

## 2. Results

### 2.1. Effect of Pae + DSS on Body Weight and Blood Glucose

**Figure 2:**
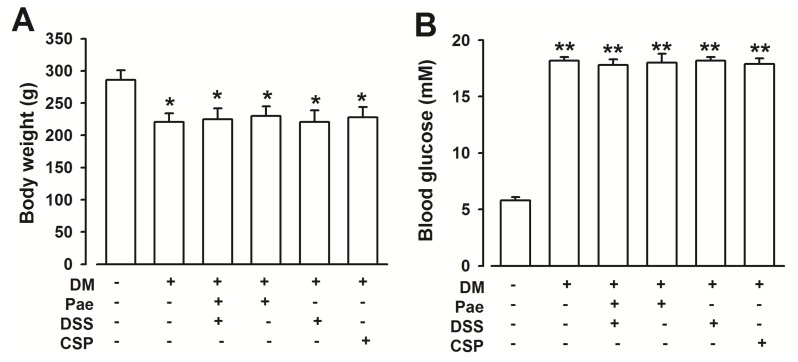
Effects of Pae + DSS and CSP (serves as a positive control) on body weight and blood glucose of diabetic rats. (**A**) Body weight significantly decreased in diabetic rats (*n* = 6) compared with that of normal rats (*n* = 20). Pae + DSS and CSP administration (*n* = 6) did not improve body weight loss in diabetic rats. (**B**) Blood glucose increased in diabetic rats (*n* = 6) compared with that of at of normal rats (*n* = 20). Pae + DSS and CSP administration (*n* = 6) did not decrease the high blood glucose in diabetic rats. *p < 0.05, **p < 0.01 compared with normal rats. DM = Diabetes Mellitus; Pae = Paeonol; DSS = Danshensu; CSP = Compound salvia pellet.

Although all rats were matched in weight at the beginning of the experiment, body weight and blood glucose did not differ between diabetic rats and diabetic rats with chronic supplementation of Pae + DSS (Figure 2A,B).

### 2.2. Activation of Pae + DSS Induced Relaxation in Rat Cerebral Artery

**Figure 3:**
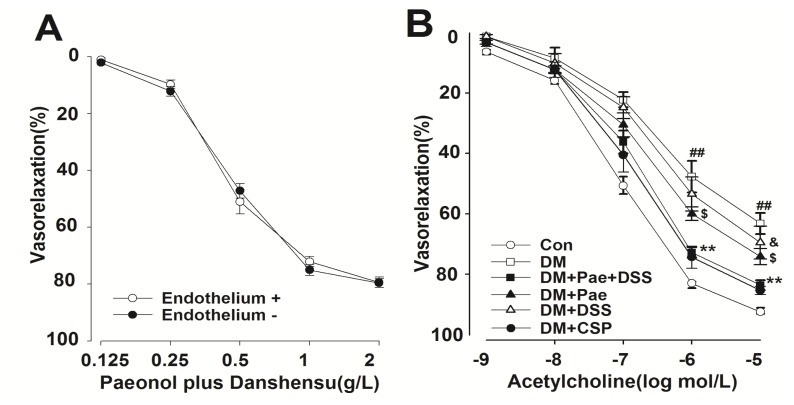
Concentration–response curves of Pae + DSS and ACh obtained in the rings of the cerebral artery of control and DM groups. (**A**) Pae + DSS induced relaxation in endothelium-intact (E+) or denuded (E−) aortic rings from normal rats (*n* = 5); (**B**) ACh-induced relaxation in all groups with functional endothelium (*n* = 6): **p < 0.01 compared with the corresponding DM group, ^##^*p* < 0.01 compared with the corresponding control group, ^$^*p* < 0.05 compared with the corresponding DM group, and ^&^*p* < 0.05 compared with the corresponding DM group. Results are given as the mean ± standard error of the mean (SEM) of three independent experiments.

Pae + DSS induced a strong relaxation on arterial rings obtained from rats in a dose-dependent manner (Figure 3A). The effect of Pae + DSS in endothelium-intact and endothelium-denuded rat arterial rings was investigated to identify the role of VSMC on Pae + DSS induced vasorelaxation. The vascular relaxation induced by Pae + DSS was not abolished in the endothelium removed rings of rats (Figure 3A).

### 2.3. Effect of Chronic Pae + DSS Administration on ACh Relaxation Response and Phenylephrine (PE) Contraction Response

When the PE-induced contraction reached a plateau, ACh (10^−9^ M to 10^−5^ M) was added cumulatively. The capability of the concentration-dependent relaxation induced by ACh, which had a maximum response of 10^−5^ M, was significantly weaker in arterial segments obtained from diabetic rats than those from normal rats (Figure 3B). After chronic administration of Pae + DSS, Pae, and DSS, the capability of ACh-induced relaxation in the arterial segments of diabetic rats was enhanced significantly. In addition, the degree of ACh-induced relaxation in the DM + Pae + DSS group was stronger than that of Pae and DSS treated diabetic rats. No marked changes were observed concerning the degree of ACh-induced relaxation between the DM + Pae + DSS group and compound salvia pellet (CSP) group.

**Figure 4:**
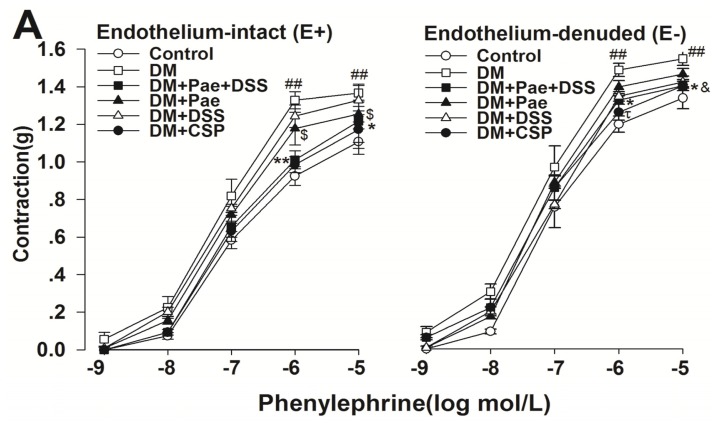

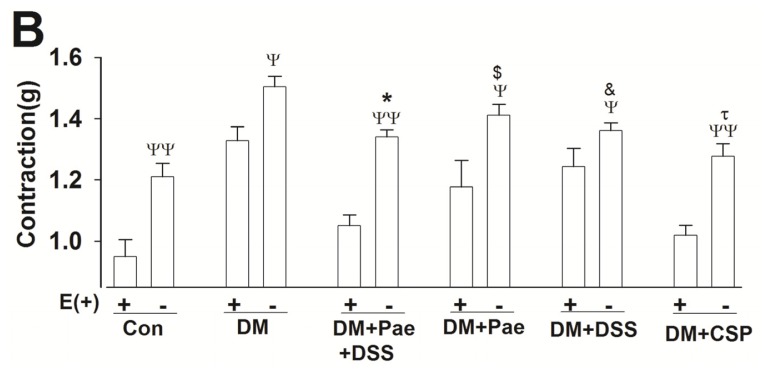
Contractile responses to *Phenylephrine* (PE) obtained from cerebral artery rings in all groups. (**A**) Concentration–response curves of PE obtained in E+ or E− rings of cerebral arteries in all groups (*n* = 5); (**B**) 1 μmol/L PE-induced contractile responses in E+ or E− rings of cerebral arteries in all groups (*n* = 5); **p < 0.01 and *p < 0.05 compared with the corresponding DM group, ^##^*p*<0.01 compared with the corresponding control group, ^$^*p* < 0.05 compared with the corresponding DM group, ^&^*p* < 0.05 compared with the corresponding DM group, and ^ψψ^*p* < 0.01 and ^ψ^*p* <0.05 compared with the corresponding E+ group. Results are given as the mean ± SEM of three independent experiments.

We also found that treatment with Pae + DSS significantly reduced the maximum contraction of rings from diabetic rats (Figure 4A). Meanwhile, the enhancement rates of contractile responses to PE in all groups were significantly different in E+ and E− rings (Figure 4A). Particularly, the enhancement rate of contractile responses in Pae + DSS treated diabetic rats was lower than that Pae and DSS treated diabetic rats (Figure 4B).

### 2.4. Influence of Pae + DSS on Calcium and Potassium Channel

**Figure 5:**
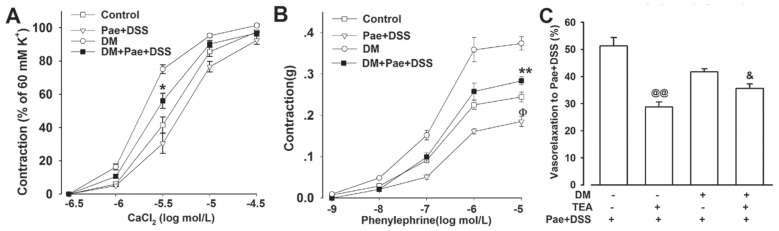
Effects of Pae + DSS on calcium channels and K^+^ channel. (**A**) Rings without endothelium from control and diabetic rats were pre-incubated with or without Pae + DSS at 0.5 g/L for 10 min; the curves of CaCl_2_ in the Ca^2+^ free solution (containing 10 M to 4 M EGTA and 60 mM KCl) were inhibited by Pae + DSS (*n* = 6); (**B**) Pre-incubation with Pae + DSS at 0.5 g/L for 10 min significantly inhibited the vasocontraction of PE in Ca^2+^ free solution containing 10 M to 4 M EGTA of rings without endothelium from control and diabetic rats (*n* = 6); (**C**) Non-selective K^+^ channel blocker tetraethylammonium (TEA, 10 mmol/L) was pre-incubated with rings from control and diabetic rats for 10 min prior to stimulation with 1 μmol/L of PE. Pae + DSS (0.5 g/L) was added after the PE contraction reached a plateau. TEA significantly reduced Pae + DSS induced relaxation. **p < 0.01 and *p <0 .05 compared with the corresponding DM group, ^Φ^*p* < 0.05 compared with the corresponding control group, ^@@^*p* < 0.05 compared with the control group treated with Pae + DSS, and ^&^*p* < 0.05 compared with the corresponding DM group treated with Pae + DSS. Results are given as the mean ± SEM of three independent experiments.

In Ca^2+^ free solutions containing KCl, pretreatment of Pae + DSS attenuated the CaCl_2_ induced contractions of denuded cerebral arteries from normal and diabetic rats (Figure 5A). In Ca^2+^-free solutions containing ethylene glycol tetraacetic acid (EGTA), Pae + DSS significantly inhibited the contractions induced by PE (Figure 5B).

To further explore the possible mechanism of the Pae + DSS induced vasoactive response, we examined the transient vasoconstrictor PE response in the presence of the non-selective K^+^ channels blocker, tetraethylammonium (TEA). In the presence of TEA, the vasorelaxation effect of Pae + DSS was partially inhibited when Pae + DSS was added after the PE contraction reached a plateau (Figure 5C).

### 2.5. Effects of Pae + DSS on SOD Activities and TBARS Content in the Cerebral Artery from Diabetic Rats

**Figure 6:**
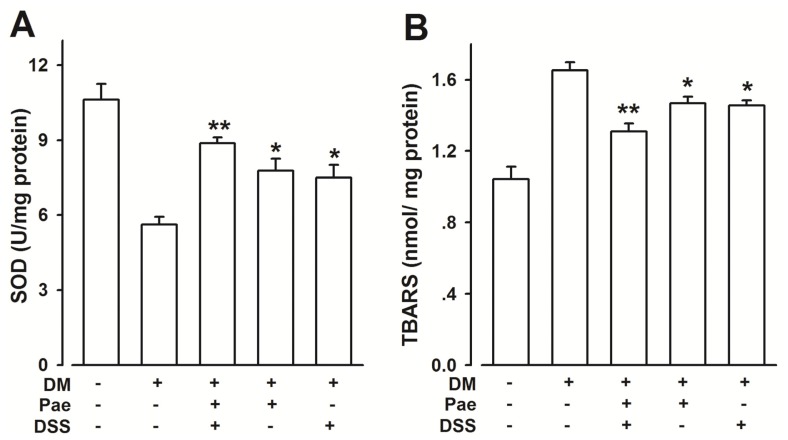
Effects of Pae + DSS on SOD activity and TBARS content from rings in all groups. The levels of SOD activity (**A**) and TBARS content (B) were assessed in the arterial tissues (*n* = 8). **p < 0.01 and *p < 0.05 compared with the corresponding DM group. Results are given as the mean ± SEM of three independent experiments.

The enhanced generation of reactive oxygen species is induced by oxidative stress. Figure 6 shows the superoxide dismutase (SOD) activities and thiobarbituric acid reactive substances (TBARS) concentrations in the arterial tissues of all groups at the end of the study. All treated groups (Pae, DSS, and Pae + DSS) exhibited increased SOD (Figure 6A) activities and decreased TBARS (Figure 6B) concentrations. Moreover, the Pae + DSS group exhibited more reduction in oxidative stress compared to Pae and DSS groups. As shown in Figure 6, administering 20 μg/kg of CSP, which was used as a positive control in the diabetic OVX rats and significantly ameliorated SOD and TBARS levels.

## 3. Discussion

Our present investigation yielded the following novel findings: (1) PE contractile responses and ACh relaxant responses in the cerebral artery of diabetic rats are partly restored by Pae, DSS, and Pae + DSS. Pae + DSS have better pharmacological effects compared with individual treatments of Pae or DSS. (2) Oxidative stress is reduced by chronic supplementation of Pae + DSS in cerebral arteries of diabetic rats. (3) *In vitro*, the combination of Pae and DSS block Ca^2+^ influx, reduce Ca^2+^ release from intracellular stores sensitive to PE in the cerebral arteries of diabetic rats and open K^+^ channels. The results suggest that Pae + DSS supplementation has the potential to improve the vascular functions of persons with diabetes.

In our study, the eight consecutive weeks of oral treatment consisted of 2.5 mg/kg of Pae, 5 mg/kg of DSS, and 1.25 mg/kg of Pae coupled with 2.5 mg/kg of DSS. Pae, DSS, and Pae + DSS did not affect the body weight of diabetic rats, and no obvious effects on blood glucose were observed. These findings suggest that Pae + DSS has no anti-diabetic effects.

Chronic oral supplementation of Pae, DSS, and Pae + DSS improved ACh relaxation and prevented enhanced PE vascular contractility in the cerebral artery (basal artery, Willis’ circle, and middle cerebral artery).

The assessment of physiologic vasodilator responses can measure endothelial dysfunction [29]. Previous studies reported that ACh-induced relaxation is significantly different in normal and diabetic groups, which suggests endothelial dysfunction [17,18,30]. Results of the present study indicate that maximum relaxations are significantly reduced in rings from diabetic rats compared with that from normal rats; however, maximum relaxation recovered partially in Pae, DSS, and Pae + DSS treated diabetic groups. Particularly, the degree of ACh-induced relaxation in Pae + DSS treated diabetic group was higher than diabetic group and all other treatment groups.

Free radical-mediated injury is considered as one of the major components involved in the pathophysiological alterations observed during DM. Furthermore, lipid peroxidation has been suggested to be closely related to diabetes-induced tissue damage. Therefore, we used TBARS (a lipid peroxidation marker) to detect the rate of lipid peroxidation. In this study, the level of TBARS of cerebral arteries from diabetic rats significantly increased and decreased with the treatment (Pae, DSS, and Pae + DSS). Meanwhile, SOD levels increased in the arterial samples from treated animals (groups DM + Pae, DM + DSS, and DM + Pae + DSS) compared with that from the DM group. In the DM + Pae + DSS group, the rate of the increase of TBARS and the decrease of SOD significantly decreased compared with those in the Pae and DSS treated diabetic groups. In addition, the results of the current study indicate that Pae + DSS induces endothelium-independent vasorelaxation in the cerebral artery of normal rats, which is not blocked in E+ rings (Figure 2A). Moreover, ACh sensitivity of cerebral arteries from diabetic rats is significantly reduced compared with that of treated diabetic rats. Medicine treatment prevented the enhanced PE vascular contractility of the cerebral artery from all treatment groups.

These results demonstrate that medicines, particularly Pae + DSS, may reduce the oxidative injury of the cerebral artery from diabetic rats. In addition, diabetic rats treated with Pae + DSS have significantly reduced oxidative injury than all the other treatment groups.

One of the major factors affecting vascular contractility is elevated myofilament Ca^2+^ sensitivity in DM [31]. Moreover, smooth muscle cells (Ca^2+^) have the potential to significantly impair normal physiological function of the vascular system [27]. Vasoconstriction is induced by different materials involved in different mechanisms. PE can induce extracellular calcium influx and PE-sensitive endogenous calcium release [32]. CaCl^2^ vasoconstriction is due to Ca^2+^ influx from the extracellular medium [33]. Therefore, CaCl^2^ and PE induced maximum contraction are significantly lower in the arterial ring of treated groups compared with those of DM groups in Ca^2+^-free medium. This result demonstrates that Ca^2+^-influx or intracellular Ca^2+^-release are reduced. Based on our results, we assume that the increased sensitivity to Ca^2+^ in DM is inhibited by chronic supplementation of Pae and DSS.

Ca^2+^ influx is well known to be inhibited by the direct activation of K^+^ channels on arterial smooth muscles [32]. After inhibition, Ca^2+^ influx triggers PE-induced vasoconstriction. In this study, TEA (non-selective K^+^ channel blocker) reduces the transient PE vasoconstrictor response more markedly in untreated groups, which suggests lower basal K^+^ channel activity in the rats.

Our previous results indicated that the content of Pae in the brain increases significantly at 10 min [15]. The pharmacological activity in cerebrovascular disease treatment can be increased by the alteration of tissue distribution in the brain. Thus, altering brain tissue distribution might enhance the therapeutic effect of the chronic supplementation of Pae + DSS.

## 4. Materials and Methods

### 4.1. Animals

Six-week-old Sprague-Dawley male rats weighing approximately 200 g were maintained on a 12 h light/dark cycle at a constant room temperature (22 °C ± 1 °C). Some of the rats were randomly divided into six groups: (1) Normal rats (Control group, *n* = 6), (2) Untreated diabetic rats (DM group, *n* = 8), (3) Diabetic rats treated with Pae (99.0% purity, Xiao Cao Botanical Development Co., Ltd., Xi’an, China) and DSS (98.5% purity, Fei Da Bio-tech. Co., Ltd., Xi’an, China) for 8 weeks (DM + Pae + DSS group, 1.25 mg/kg Pae coupled with 2.5 mg/kg DSS, orally, two times per day, *n* = 8), (4) diabetic rats treated with Pae for 8 weeks (DM + Pae group, 2.5 mg/kg, orally, two times per day, *n* = 8), (5) diabetic rats treated with DSS for 8 weeks (DM + DSS group, 5 mg/kg, orally, two times per day, *n* = 8), and (6) diabetic rats treated with compound salvia pellet (CSP, serves as positive control [34–37]) for 8 weeks (CSP group, 20 μg/kg, orally, two times per day, *n* = 8). The other (*n* = 6) rats were used to detect the acute effect of Pae + DSS (Pae:DSS = 1:2, g/L) on the basal tonus of the artery ring. Pae and DSS were suspended in a 0.3% CMC-Na solution.

Type 2 diabetic rats were fed with a high-fat diet (HFD) for 2 months and given low doses of streptozotocin (STZ, 30 mg/kg i.p.; Sigma-Aldrich, St. Louis, MO, USA), which was dissolved in a sodium citrate buffer (pH 4.2).

In the control group, the same volume of sodium citrate buffer was injected intraperitoneally. The remaining rats were fed with normal rodent diet, which were also injected with a similar volume of citrate buffer. The composition of the normal rodent’s diet was 20% protein and 4.5% fat. The HFD diet composed of 21.2% protein, 12% fat, 15% sucrose, and 1% cholesterol. Blood glucose concentrations were measured by a glucometer (Accutrend; Bayer, Mannheim, Germany) 48 h post-STZ injection. Rats with blood glucose ≥13 mM were considered diabetic. The current study was performed in adherence to the National Institutes of Health guidelines for the use of experimental animals. All animal protocols were approved by the Committee for Ethical Use of Experimental Animals of the Fourth Military Medical University.

### 4.2. Vascular Reactivity

The animals were anesthetized via intraperitoneal administration of 20% urethane 8 weeks post-injection of STZ or buffer. The rat’s cerebral artery (basal artery, Willis’ circle, and middle cerebral artery) was removed and placed in an ice-cold Krebs buffer consisting of the following: 118 mM NaCl, 4.8 mM KCl, 2.5 mM CaCl_2_·2H_2_O, 2.5 mM MgCl_2_·6H_2_O, 1.2 mM NaH_2_PO_4_·2H_2_O, 8.5 mM NaHCO_3_, and 11 mM glucose·H_2_O. The cerebral artery was cleared of fat as well as connective tissue and cut into 2 mm-long rings. The rings were mounted onto hooks, suspended in organ chambers filled with Krebs buffer, aerated with 95% O_2_ + 5% CO_2_ at 37 °C, and connected to pressure transducers (WPI, Sarasota, FL, USA) to record changes via Mac-Lab recording system. After 30 min of equilibration at an optimal preload of 9.8 mN, the rings were stimulated with 10^−6^ mol/L of PE and 10^−6^ mol/L of ACh (both from Sigma-Aldrich, St. Louis, MO, USA). Rings with >50% relaxation were considered E+.

### 4.3. Effect of Pae + DSS on the Basal Tonus of Arterial Rings

After equilibration, E+ and E− rings of the normal rats were incubated with various concentrations of Pae + DSS (0.125 g/L to 2 g/L). Afterward, the concentration–response curves for Pae + DSS was determined.

### 4.4. Effect of Chronic Pae + DSS Administration on the Relaxation Response to ACh and the Contraction Response to PE

To investigate the role of Pae + DSS on vascular functions, diabetic rats were treated with Pae + DSS, Pae, and DSS for eight weeks. The cerebral arteries from all groups were preconstricted with PE in organ chambers eight weeks later. When the PE-induced contraction had reached a plateau, ACh (10^−9^ M to 10^−5^ M) was added cumulatively. Subsequently, to further clarify the role of Pae + DSS on the vascular function of diabetic rats, E+ and E- contractions to PE were studied in cerebral artery.

### 4.5. Effect of Chronic Pae + DSS Administration on the Contraction Response to PE and CaCl_2_ in Ca^2+^-Free Solution

To investigate whether the chronic supplementation of Pae + DSS could interfere with Ca^2+^ release from intracellular stores and whether the effect was stronger than Pae or DSS groups, a Ca^2+^-free solution containing EGTA (1 mmol/L) replaced the normal K–H solution. The E− rings were exposed to a Ca^2+^-free solution for 20 min and then stimulated with PE (1 μM) in the organ chambers. To further clarify the role of Pae + DSS on extracellular calcium influx, E- rings contracted with PE was made to deplete the intracellular Ca*^2+^* stores and maintained in status for 50 min. The E− rings were then rinsed in a Ca^2+^-free solution without EGTA containing KCl (60 mmol/L). Afterward, cumulative concentration–response curves for CaCl*_2_* (ranging from 10^−6.5^ M to 10^−4.5^ M) can be determined in E− rings.

### 4.6. Effect of K^+^ Channel Blockers on Pae + DSS Induced Vascular Reactivity

To further clarify the possible mechanisms responsible for Pae + DSS induced vascular reactivity, E− rings from control and diabetic rats were pre-incubated with TEA (10 mmol/L) for 10 min before PE was added.

### 4.7. SOD Activity Assay and TBARS Level

Tissue samples of rat cerebral artery were homogenized in a phosphate buffer (1/10 *w*/*v*; pH = 7.0), and then centrifuged for 20 min at 10,000× rpm/min at 4 °C. Determination of SOD activities in the supernatants were carried out immediately. The activity levels of SOD were determined according to the kit specifications of a visible light photometer (Nanjing Jiancheng Bioengineering Institute, Nanjing, China). Lipid peroxidation was briefly measured by the determination of TBARS (a lipid peroxidation marker, Hayward, CA, USA), which was performed according to the instructions of QuantiChromTM TBARS Assay Kit (QuantiChrom, BioAssay Systems, Hayward, CA, USA).

### 4.8. Statistical Analysis

Data are expressed as mean ± SEM. Statistical comparisons were performed using *t*-test. Differences between multiple groups were assessed using one-way analysis of variance. *p* < 0.05 was considered statistically significant.

## 5. Conclusions

Our findings indicate that the combined treatment with Pae and DSS improves vascular dysfunction in diabetes compared with individual treatments of Pae or DSS. This study further demonstrate that the observed cerebroprotective effect from Pae+DSS treated cerebral arteries in diabetes was due to two complementary mechanisms: decreased stress-induced vascular injury during DM and attenuated intracellular calcium concentration through the promotion of non-selective K^+^ channel opening, which impairs both Ca^2+^ influx and Ca^2+^ release.
